# Supplementary material for: Rapid evolution of an adaptive multicellular morphology of Candida auris during systemic infection
Source: Nat Commun. 2024 Mar 16;15:2381. doi: 10.1038/s41467-024-46786-8 (PMC10944540; doi:10.1038/s41467-024-46786-8)
Supplement: Supplementary file 1 — Supplementary Information [file 41467_2024_46786_MOESM1_ESM.pdf]

Supplementary Information for

Rapid evolution of an adaptive multicellular morphology of *Candida auris* during systemic infection

Jian Bing[1,2,#], Zhangyue Guan[1,#], Tianhong Zheng[1,2], Craig L. Ennis[3,4], Clarissa J. Nobile[3,5], Changbin Chen[6], Haiqing Chu[7,8,*], and Guanghua Huang[1,2*]

*Please direct all correspondence to:

Haiqing Chu

Department of Respiratory and Critical Care Medicine, Shanghai Pulmonary Hospital, School of Medicine, Tongji University, Shanghai, China, E-mail: chu_haiqing@126.com

Guanghua Huang (ORCiD: 0000-0002-4761-7548)

State Key Laboratory of Genetic Engineering, School of Life Sciences, Fudan University, Shanghai 200438, China

Tel: 86-21-31246792 Fax: 86-21-31246792 Email: huanggh@fudan.edu.cn

This PDF file includes:

Supplementary Figures S1 to S13, Tables S1 to S4

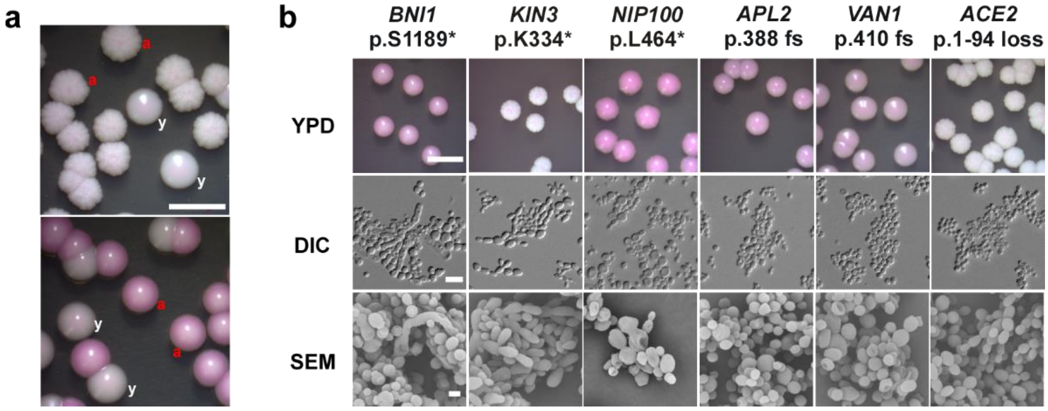

Supplementary Figure 1. Colony and cellular morphologies of six additional experimentally evolved *C. auris* aggregative strains. (a) Representative colony morphologies of aggregative strains. (b) *BNI1* p.S1189* (FDAG44), *KIN3* p.K334* (FDAG42), *NIP100* p.L464* (FDAG62), *APL2* p.388fs (FDAG5), *VAN1* p.410fs (FDAG28), and *ACE2* p.1-94 loss (FDAG37) represent six evolved strains with mutations in *BNI1, KIN3*, *NIP100, APL2, VAN1, and ACE2*, respectively. *C. auris* cells were plated onto YPD plates supplemented with phloxine B at 30°C for 4 days. *, nonsense mutations; fs, frameshift mutations; loss, missing partial ORF region. Scale bar for colony, 5 mm; for DIC morphology, 10 μm; for SEM and TEM morphologies, 2 μm. The mutant strains were isolated from the infected mouse tissues. This figure is associated with **Fig. 2** and **Fig. 3**.

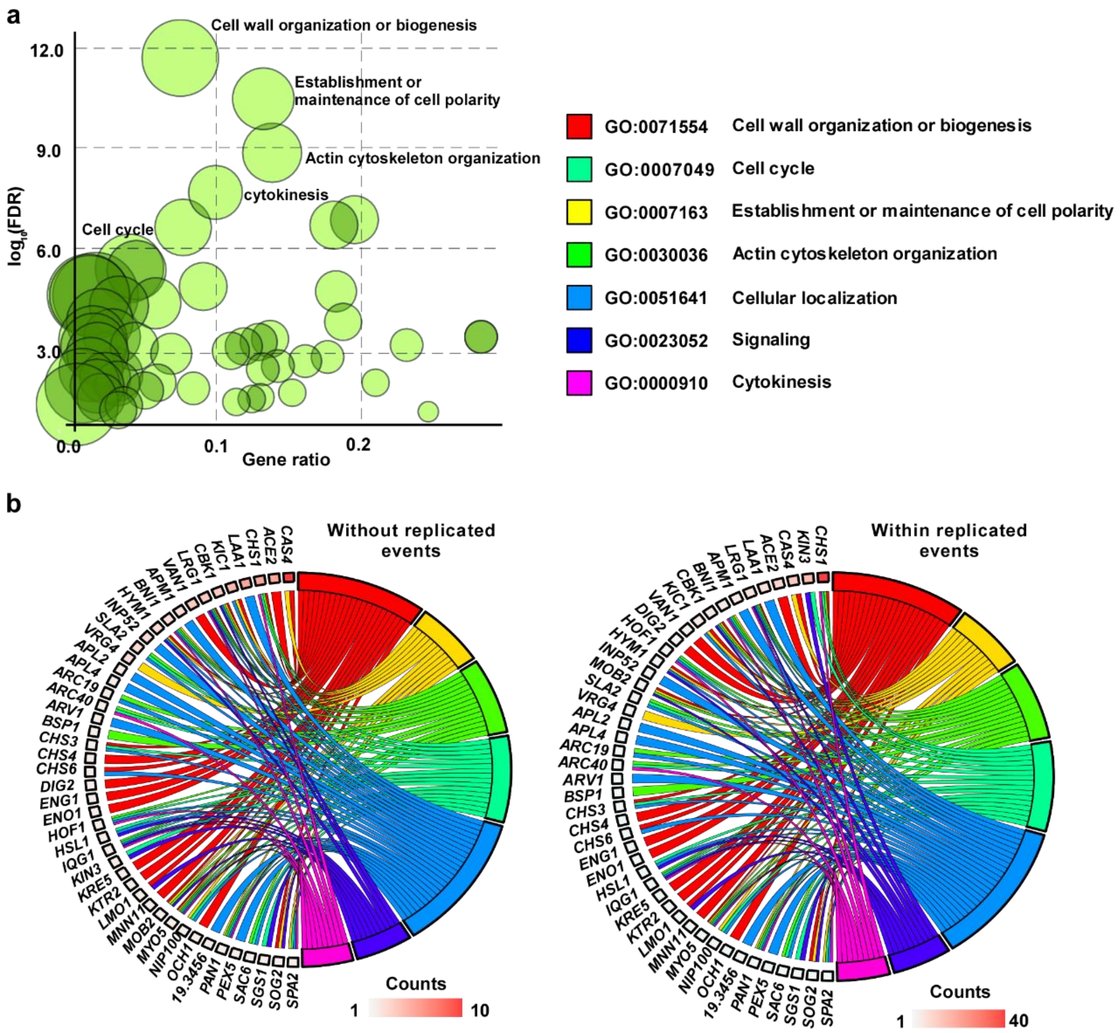

Supplementary Figure 2. Gene ontology (GO) enrichment analysis of mutated genes associated with the evolved aggregative *C. auris* strains. (a) Bubble plot of GO enrichment of 45 genes identified from the aggregative mutant strains. The x-axis represents the gene ratio for the specific term indicated. The y-axis represents the log_2 adjusted p-value. The bubble size represents enrichment gene counts. (b) Chord plot of 7 GO categories. The left panel shows the gene counts without replicated genotypes and the right panel shows the gene counts with replicated genotypes.

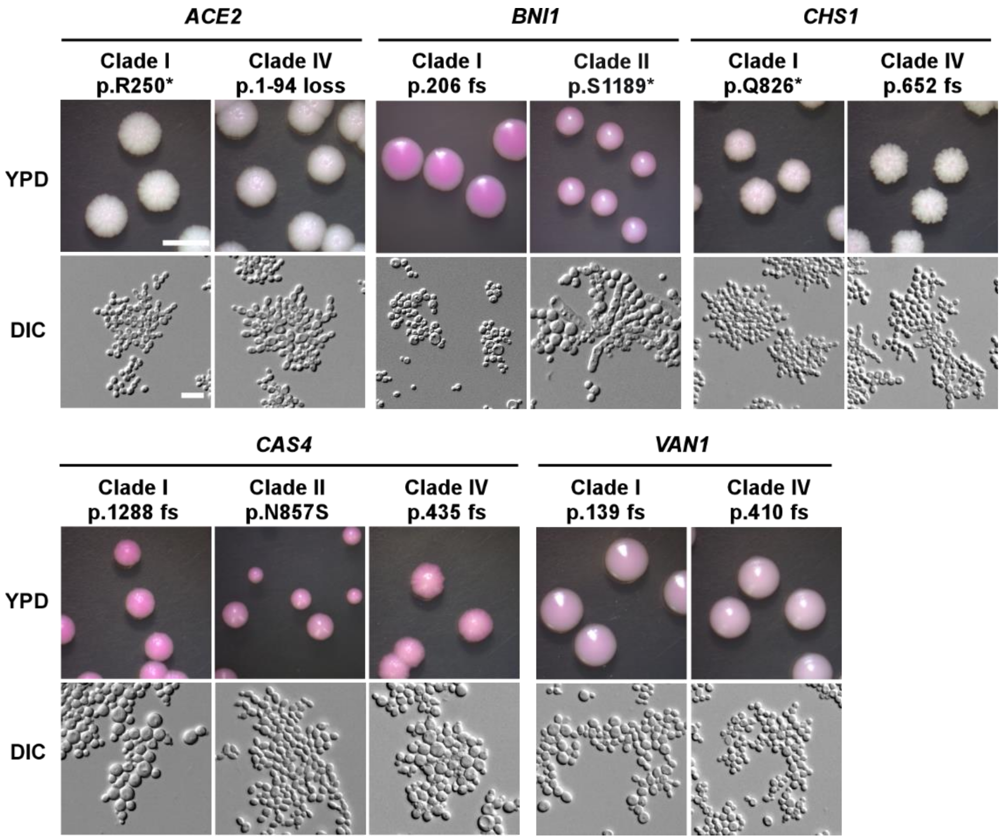

Supplementary Figure 3. Colony and cellular morphologies of evolved aggregative mutants of *ACE2, BNI1, CAS4, CHS1*, and *VAN1* genes derived from different parental strains. Strains used: *ACE2* p.R250* (clade I, FDAG36), *ACE2* p.1-94 loss (clade IV, FDAG37), *BNI1* p.206fs (clade I, FDAG30), *BNI1* p.S1189* (clade II, FDAG44), *CAS4* p.1288fs (clade I, FDAG35), *CAS4* p.N857S (clade II, FDAG50), *CAS4* p.435fs (clade IV, FDAG38), *CHS1* p.Q826* (clade I, FDAG4), *CHS1* p.652fs (clade IV, FDAG13), *VAN1* p.139fs (clade I, FDAG11), *VAN1* p.410fs (clade IV, FDAG28). *C. auris* cells were plated onto YPD plates supplemented with phloxine B at 30°C for four days. *, nonsense mutations; fs, frameshift mutations; loss, loss of partial ORF region. Scale bar for colony, 5 mm; for cell morphology, 10 μm.

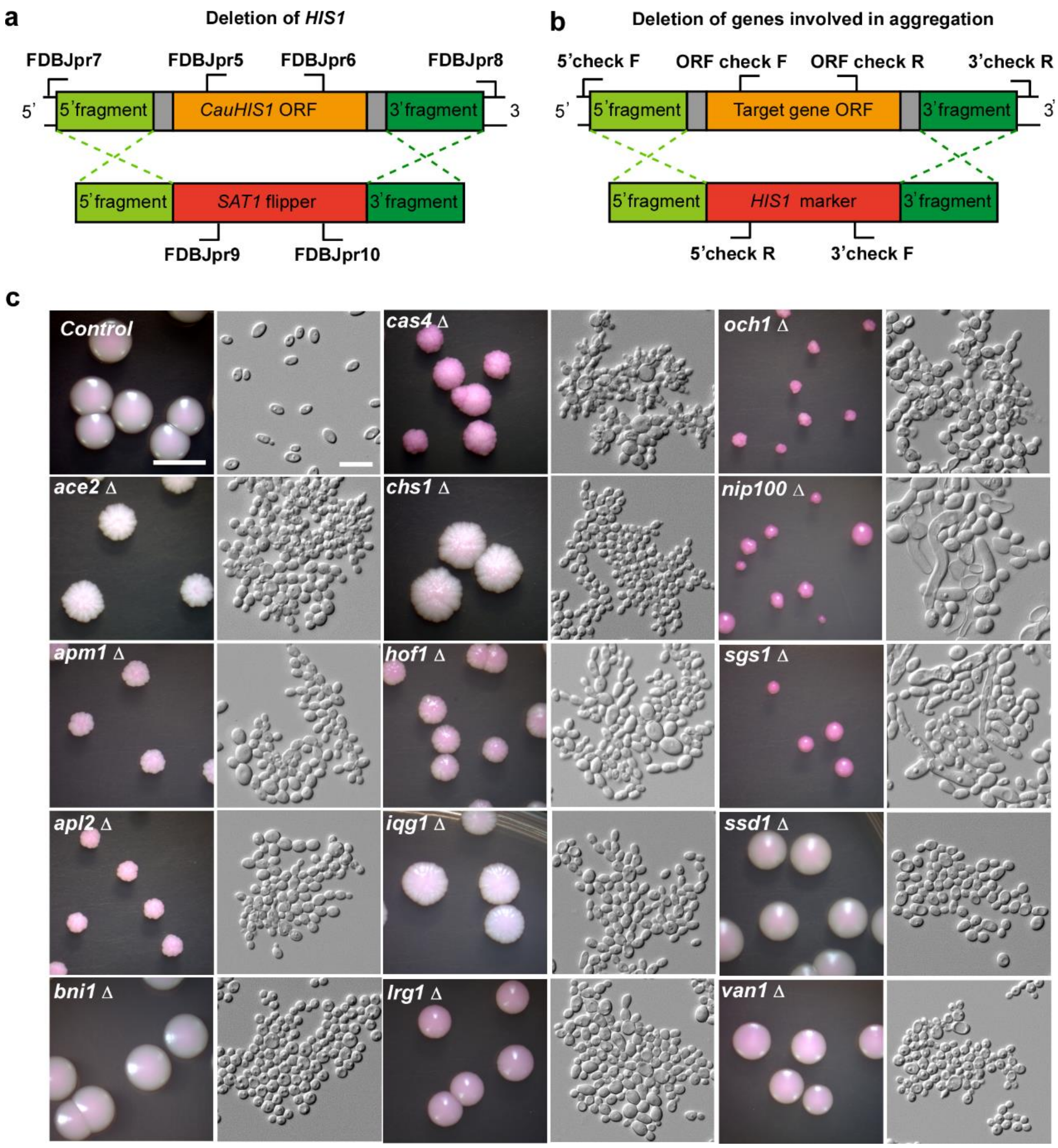

Supplementary Figure 4. Colony and cellular morphologies of additional constructed deletion mutant strains. (a) Schematic of *HIS1* deletion in *C. auris* (BJCA001). Plasmid PSFS2A was used for deletion (see Methods). The *caSAT1* flipper cassette was excised by growth in YPM (YP + maltose) medium. Primers used for checking of deleted genes are indicated. (b) Schematic of *C. auris* gene deletion using the *CauHIS1* selectable marker and *Cauhis1*Δ strain. Primers used for checking deleted genes are indicated. (c) Colony and cellular morphologies of *C. auris* gene deletion mutants. The strains used are listed in

supplementary **Dataset S1**. All strains were derived from strain FSR1319. Strains used: Control, (*HIS1*-reconstituted, FDAG292), *ace2-* (FDBJ329), *cas4-* (FDBJ288), *chs1-* (FDBJ282), *bni1-* (FDBJ286), *hof1-* (FDBJ358), *iqg1-* (FDBJ354), *apl2-* (FDBJ295), *apm1-* (FDBJ350), *irg1-* (FDBJ333), *ssd1-* (FDBJ339), *van1-* (FDBJ334), *och1-* (FDBJ366), *nip100-* (FDBJ370), and *sgs1-* (FDBJ362). Fungal cells were plated and grown onto YPD plates containing phloxine B at 30°C for 4 days. Scale bars for colony, 5 mm; for cells, 10 μm.

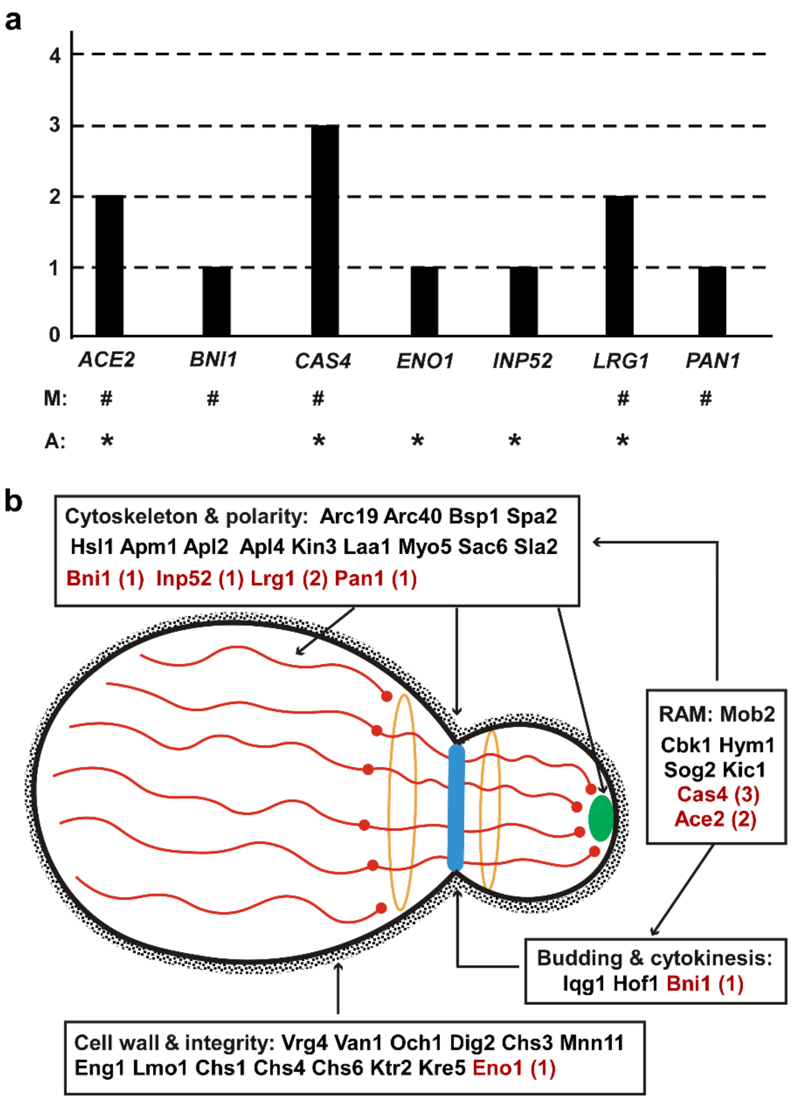

Supplementary Figure 5. Identification of gene mutations that are potentially associated with the aggregative phenotype in *C. auris* from analysis of the genomic data of clinical *C. auris* isolates deposited in the NCBI database. (a) Number of genomes containing variations of each gene. Mutations (including nonsense and frameshift, grey) were analyzed. “#” indicates mutated genes screened in the mouse systemic infection assays (M) and “*” indicates mutated genes screened in the antimicrobial peptide treatment assays (A) in our study. (b) Schematic model depicting the identified mutated genes involved in aggregate formation. All evolved strains isolated from the

mouse systemic infection model and antimicrobial peptide killing assays in this study are shown. Genes highlighted in red were identified based on the *C. auris* genomic sequences available in the NCBI database.

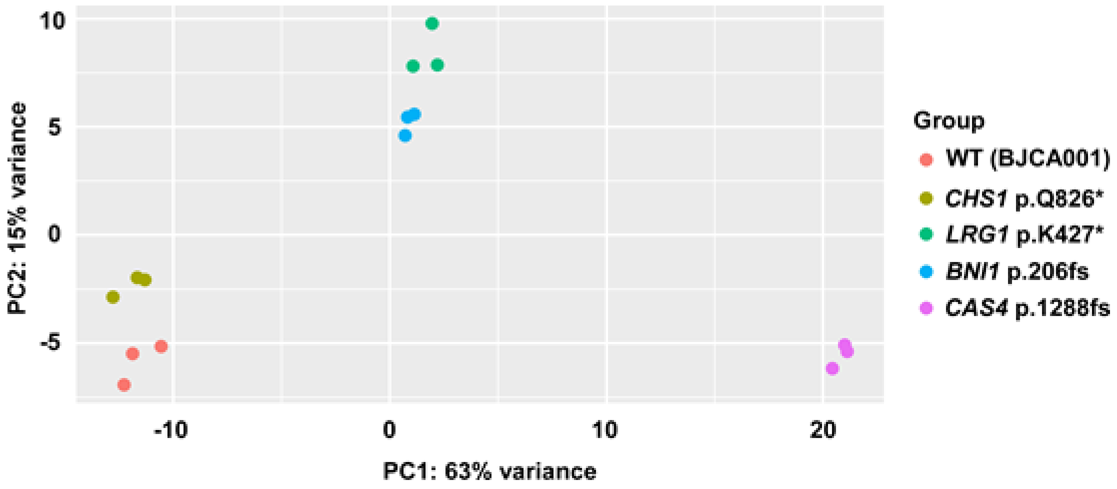

Supplementary Figure 6. Principal component analysis (PCA) of the 15 transcriptome samples. Strains used: BJCA001 (yeast-form, red), *CHS1* p.Q826* (FDAG4, chartreuse), *BNI1* p.206fs (FDAG30, blue), *LRG1* p.K427* (FDAG9, green), and *CAS4* p.1288fs (FDAG1, purple). Three biological repeats were performed for each strain.

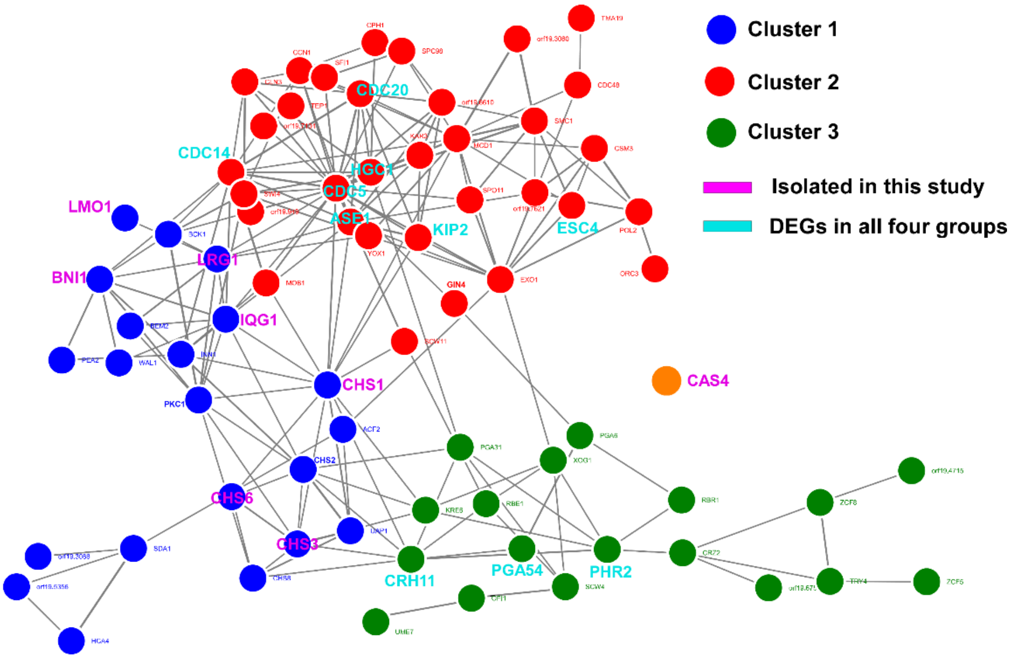

Supplementary Figure 7. The regulatory network of DEGs between the *CHS1*p.Q826* (FDAG4), *BNI1*p.206fs (FDAG30), *LRG1*p.K427* (FDAG9), and *CAS4*p.1288fs (FDAG1) mutant strains and the yeast-form strain (BJCA001). 106 DEGs were found in at least two comparative groups (yeast-form strain/*CHS1*p.Q826* mutant strain, yeast-form strain/*BNI1*p.206fs mutant strain, yeast-form strain/*LRG1*p.K427* mutant strain, and yeast-form strain/*CAS4*p.1288fs mutant strain), which are associated with the regulation of cytoskeleton, cell cycle/cytokinesis, cell wall integrity, and cell polarity processes based on by the STRING web tool. Grey lines between target proteins (red, blue, and green cycles) indicate the connections (interactors). Three clusters were predicted using K-means clustering. Genes regulating the aggregative phenotype identified in this study were highlighted in purple. Common DEGs identified in the four mutant strains were highlighted in cyan.

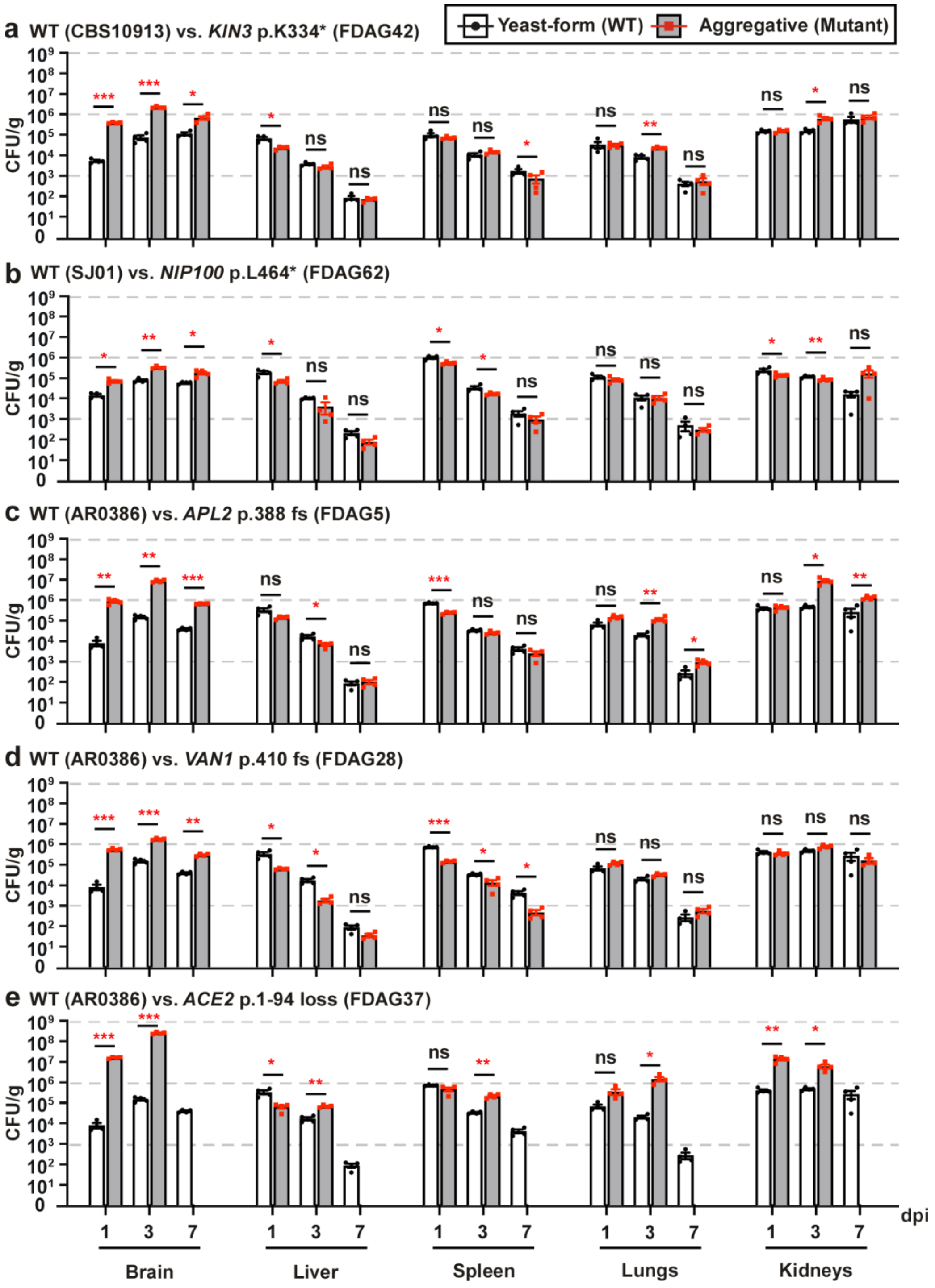

Supplementary Figure 8. Comparative analyses of fitness and virulence of the yeast-form and aggregative cells using a competing mouse systemic infection model. (a-e) CFU assays (per gram organ) were performed at 1, 3, and 7 dpi and fungal burdens of the WT and evolved mutant strains in different mouse organs are shown. Four mice per strain were

examined at each time point. (a) WT strain (yeast-form, CBS10913) versus *KIN3* p.K334* (FDAG42); (b) WT strain (yeast-form, SJ2) versus *NIP100* p.L464* (FDAG62); (c) WT strain (yeast-form, AR0386) versus the *apl2* mutant strain *APL2* p.388fs (FDAG5); (d) WT strain (yeast-form, AR0386) versus the *van1* mutant strain *VAN1* p.410fs (FDAG28); (e) WT strain (yeast-form, AR0386) versus *ACE2* p.1-94 loss (FDAG37). The *P* value was determined by two-tailed paired Student's *t*-tests. $^{*}P < 0.05$; $^{**}P < 0.01$; $^{***}P < 0.001$. Data shown represents mean ± SD.

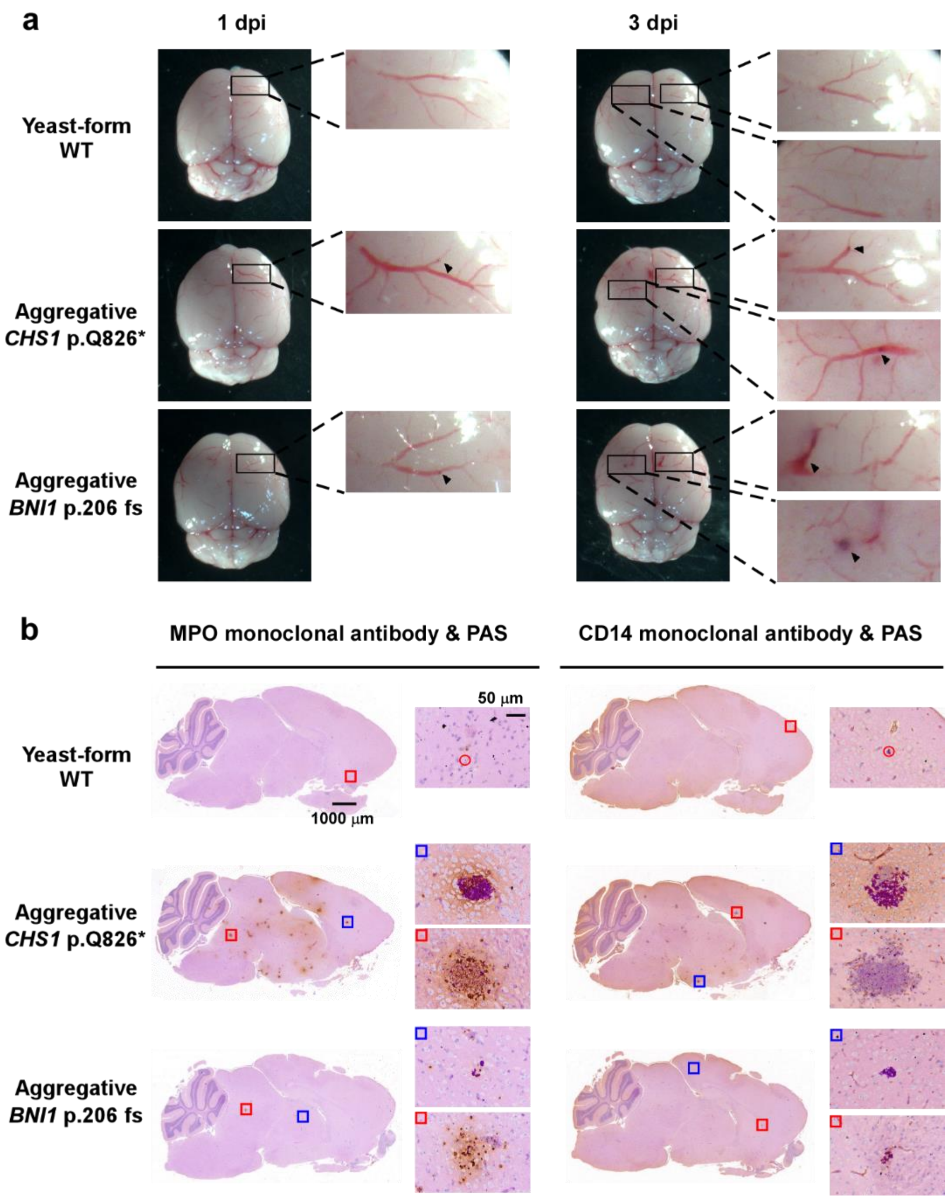

Supplementary Figure 9. *C. auris* aggregative cells cause more severe damage and have increased host innate immune responses in the brain compared to yeast-form cells. (a) Brain lesions caused by *C. auris* infections. Black arrows indicate telangiectasia or tissue lesions as seen as dark spots or accumulated blood. (b) Periodic acid-Schiff (PAS) staining and immunohistochemistry assays. Monoclonal antibodies to MPO and CD14 were used to reveal monocytes and neutrophils. Whole brain images were created using panoramic scan imaging. Strains used: WT (yeast-form, BJCA001), *CHS1* p.Q826* (FDAG4) and *BNI1* p.206fs (FDAG30).

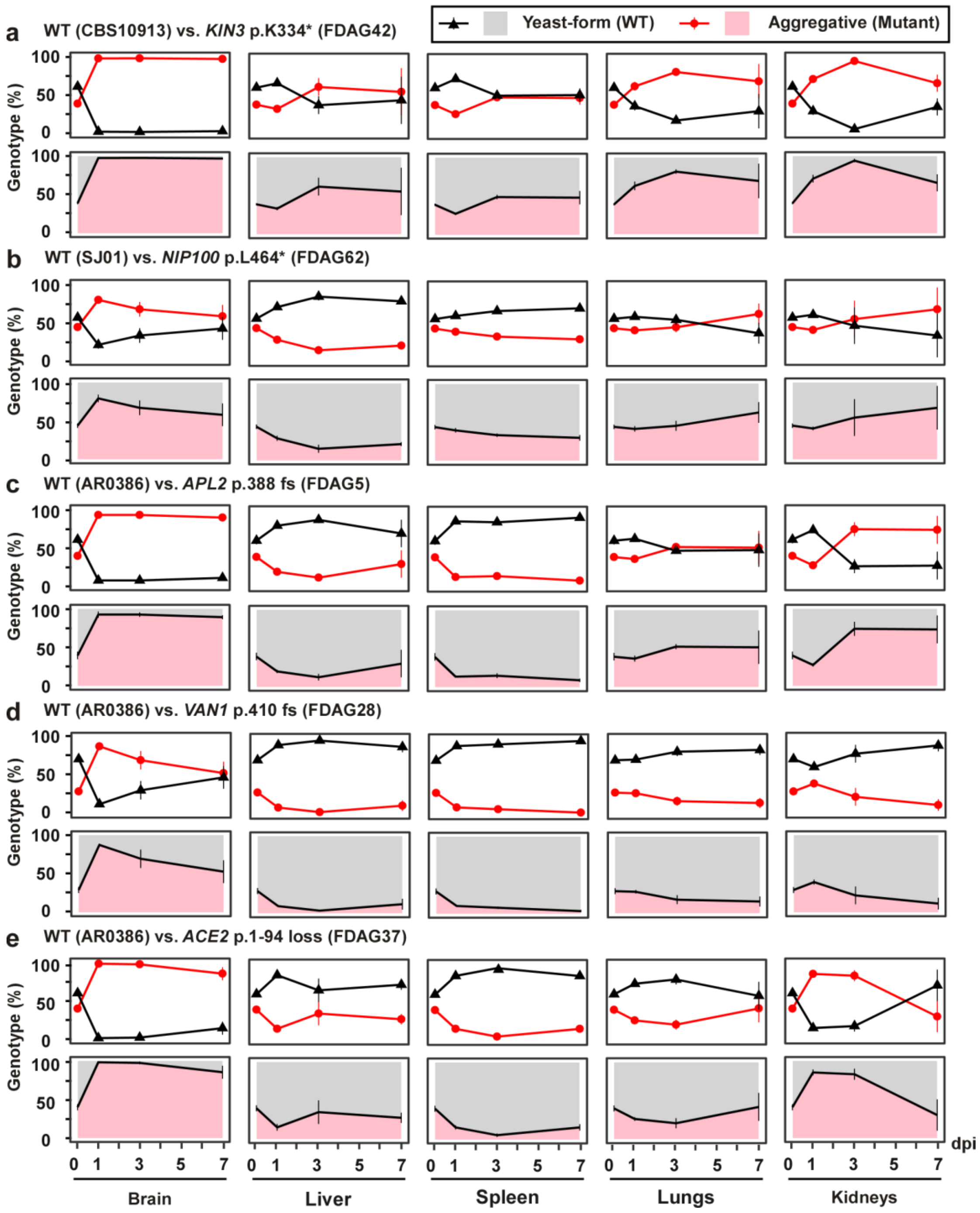

Supplementary Figure 10. Competitive fitness and virulence assays for *C. auris* yeast-form and aggregative cells. (a-e) Equal numbers of single cells of the two morphologies (5×10^6 yeast-form cells + 5×10^6 aggregative cells) were mixed and injected into the mice via the tail vein. At 1, 3, or 7 dpi, fungal cells were recovered from the brain, liver, spleen, lung, and kidney and re-

plated onto YPD medium plates containing the red dye phloxine B. CFU assays (per gram organ) were performed. Four mice per strain were examined at each time point. (a-e) Percentages of the yeast-form (WT) and aggregative (evolved mutant) cells in different mouse organs based on CFU assays. (a) WT strain (yeast-form, CBS10913) versus *KIN3* p.K334* (FDAG42); (b) WT strain (yeast-form, SJ2) versus *NIP100* p.L464* (FDAG62); (c) WT strain (yeast-form, AR0386) versus the *apl2* mutant strain *APL2* p.388fs (FDAG5); (d) WT strain (yeast-form, AR0386) versus the *van1* mutant strain *VAN1* p.410fs (FDAG28); (e) WT strain (yeast-form, AR0386) versus *ACE2* p.1-94 loss (FDAG37). Data shown represents mean ± SD.

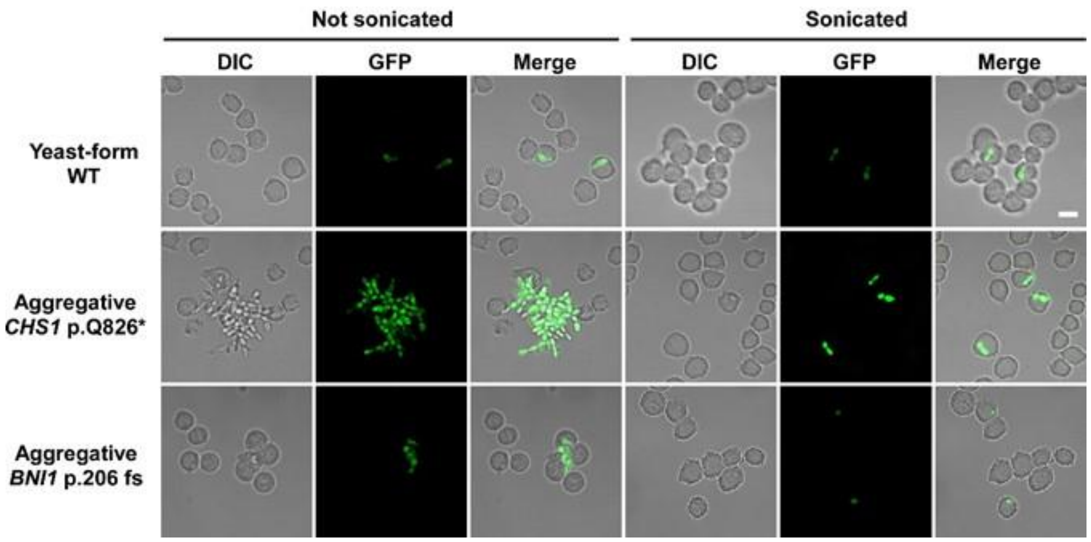

Supplementary Figure 11. Macrophage cells are unable to engulf *C. auris* cell aggregates. One yeast-form strain (GZY121) and two representative aggregative mutant strains *CHS1* p.Q826* (GZY127) and *BNI1* p.206fs (GZY144) were tested. *C. auris* cells of these strains carried a GFP reporter under the control of the strong *TDH3* promoter. To uncover whether cell aggregation (but not other biological characteristics) caused the reduced susceptibility to macrophages, we examined both the sonication-treated and sonication-untreated fungal cells. Sonication treatment converted aggregative cells to single cells. *C. auris* cells were co-incubated with the macrophage cell line RAW264.7 for one hour and subjected to confocal scanning laser microscopy (CSLM) assays. Scale bar, 10 μm.

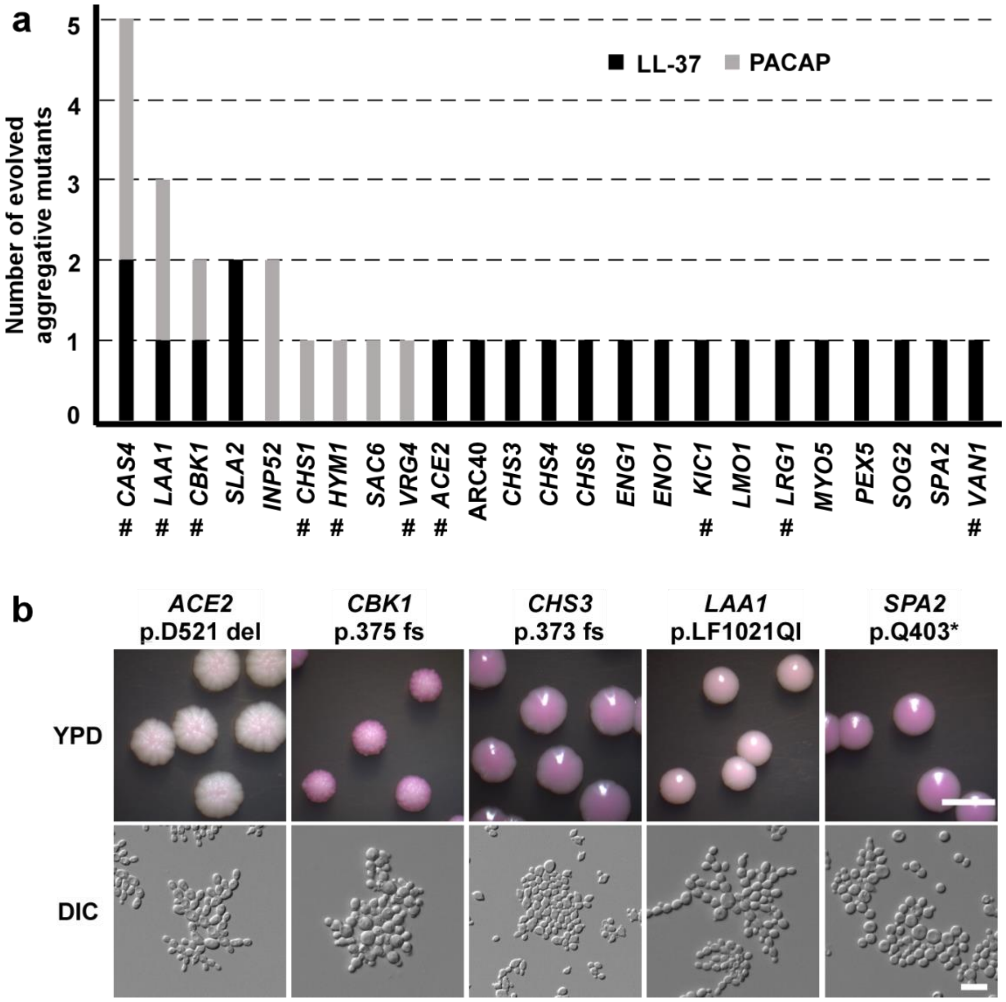

Supplementary Figure 12. Isolation of evolved aggregative strains after exposure to host antimicrobial peptides. (a) Numbers of isolates for each mutated gene. 1×10^6 yeast-form cells of *C. auris* were resuspended in 1 mM PPB and treated with 5 μM LL-37 (black) or 1 μM PACAP (grey) for 1 hour at 37°C. The treated cells were then diluted and plated onto YPD medium plates containing phloxine B. Wrinkled or red/pink colonies were picked for microscopy assays and whole genome sequencing (WGS) analysis. "#" indicates mutated genes also identified in the mouse systemic infection assays in **Fig. 3a**. (b) Colony and cellular morphologies of representative aggregative strains induced

by the host antimicrobial peptides. Strain used: *ACE2* p.D521 del (FDAG104), *CBK1* p.375fs (FDAG101), *CHS3* p.373fs (FDAG109), *LAA1* p.LF1021QI (FDAG114), *SPA2* p.Q403* (FDAG103). *C. auris* cells were plated onto YPD plates supplemented with phloxine B at 30°C for 4 days. “*” indicates nonsense mutations; fs indicates frameshift mutations; “del” indicates sequence deletion mutations. Scale bar for colony, 5 mm; for cell morphology, 10 μm.

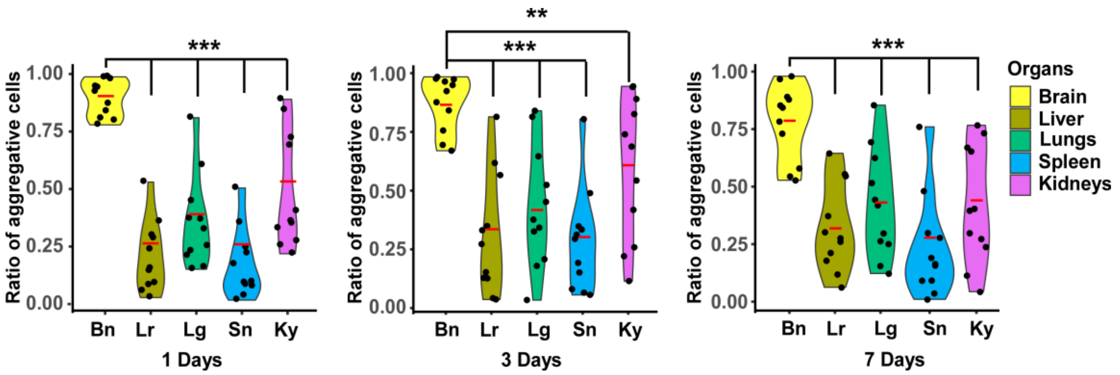

Supplementary Figure 13. Aggregative cells of *C. auris* have a higher fungal burden in systemic infections. Summary of the percentages of aggregative cells in different organs in the competitive mouse infection model for 10 evolved aggregative mutant strains. The 10 infection groups are WT (AR0386) + *SSD1* p.R549K (AR0386A), WT (BJCA001) + *CHS1* p.Q826* (FDAG4); WT (BJCA001) + *BNI1* p.206fs (FDAG30), WT (BJCA001) + *LRG1* p.K427* (FDAG9), WT (BJCA001) + *CAS4* p.1288fs (FDAG1), WT (CBS10913) + *KIN3* p.K334* (FDAG42), WT (SJ01) + *NIP100* p.L464* (FDAG62), WT (AR0386) + *APL2* p.388fs (FDAG5), WT (AR0386) + *VAN1* p.410fs (FDAG28), and WT (AR0386) + *ACE2* p.1-94 loss (FDAG37). Bn, brain; Lr, livers; Sn, spleens; Lg, lungs; Ky, kidneys. Red lines indicate the average values. Two-tailed paired Student's *t*-tests were used to assess significance. The *P* value was determined by two-tailed paired Student's *t*-tests. *$P < 0.05$; **$P < 0.01$; ***$P < 0.001$. Data shown represents mean ± SD. This figure summarizes the results of **Fig. 6** and **Fig. S10**.

Table S1. Isolated aggregated mutant strains from the mouse infection system.

No.	Parental strain	Reference or source	Number of Infected mice	Number of each evolved mutant	Name of evolved isolate	Genotype	Used in this study
1	BJCA001 (Clade I)	Wang et al. 2018[1]	4	53	FDAG36	ACE2 p.R250*	Figure S3
					FDAG3	ACE2 p.K254*	
					FDAG30	BNI1 p.206fs	Figure 2,4,5, 6,8,S3,S7,S9,S11
					FDAG35	CAS4 p.Y203*	
					FDAG1	CAS4 p.1288fs	Figure 2,4, 5,6,8,S3,S7
					FDAG2	CAS4 p.1288fs	
					FDAG4	CHS1 p.Q826*	Figure 2,4,5, 6,8,S3,S7,S9,S11
					FDAG14	CHS1 p.Q826*	
					FDAG17	CHS1 p.Q826*	
					FDAG18	CHS1 p.Q826*	
					FDAG19	CHS1 p.Q826*	
					FDAG20	CHS1 p.Q826*	
					FDAG21	CHS1 p.Q826*	
					FDAG26	CHS1 p.Q826*	
					FDAG31	CHS1 p.Q826*	
					FDAG34	CHS1 p.Q826*	
					FDAG71	CHS1 p.Q826*	
					FDAG72	CHS1 p.Q826*	
					FDAG73	CHS1 p.Q826*	
					FDAG74	CHS1 p.Q826*	
					FDAG75	CHS1 p.Q826*	
					FDAG76	CHS1 p.Q826*	
					FDAG77	CHS1 p.Q826*	
					FDAG78	CHS1 p.Q826*	
					FDAG79	CHS1 p.Q826*	
					FDAG80	CHS1 p.Q826*	
					FDAG81	CHS1 p.Q826*	
					FDAG82	CHS1 p.Q826*	
					FDAG83	CHS1 p.Q826*	
					FDAG84	CHS1 p.Q826*	
					FDAG85	CHS1 p.Q826*	
					FDAG86	CHS1 p.Q826*	
					FDAG87	CHS1 p.Q826*	

					FDAG88	CHS1 p.Q826*	
					FDAG89	CHS1 p.Q826*	
					FDAG90	CHS1 p.Q826*	
					FDAG91	CHS1 p.Q826*	
					FDAG92	CHS1 p.Q826*	
					FDAG93	CHS1 p.Q826*	
					FDAG94	CHS1 p.Q826*	
					FDAG32	CHS1 p.G557*	
					FDAG33	KIC1 p.716fs	
					FDAG25	KIC1 p.L79H	
					FDAG27	KIC1 p.745fs	
					FDAG98	LAA1 p.1858fs	
					FDAG67	LAA1 p.D2106N	
					FDAG68	LAA1 p.D2106N	
					FDAG8	LRG1 p.K427*	
					FDAG9	LRG1 p.K427*	Figure 2,4, 5,6,8,S7
					FDAG10	LRG1 p.K427*	
					FDAG22	LRG1 p.K427*	
					FDAG40	OCH1 p.Q204*	
					FDAG11	VAN1 p.139fs	Figure S3
2	CBS10913 (Clade II)	CBS-KNAW culture collection	4	18	FDAG44	BNI1 p.S1189*	Figure S1,S3
					FDAG59	BNI1 p.S1189*	
					FDAG60	BNI1 p.S1189*	
					FDAG42	KIN3 p.K334*	Figure S1,S8,S10
					FDAG43	KIN3 p.K334*	
					FDAG45	KIN3 p.K334*	
					FDAG46	KIN3 p.K334*	
					FDAG47	KIN3 p.K334*	
					FDAG48	KIN3 p.K334*	
					FDAG49	KIN3 p.K334*	
					FDAG51	KIN3 p.K334*	
					FDAG52	KIN3 p.K334*	
					FDAG55	KIN3 p.K334*	
					FDAG56	KIN3 p.K334*	
					FDAG57	KIN3 p.K334*	
					FDAG65	KIN3 p.K334*	
					FDAG66	KIN3 p.K334*	
					FDAG29	PAN1 p.743fs	
3	CBS12372 (Clade II)	CBS-KNAW	2	3	FDAG70	BSP1 loss	
					FDAG50	CAS4	Figure S3

		culture collection				p.N857S	
					FDAG54	HSL1 p.1202fs	
4	CBS12373 (Clade II)	CBS-KNAW culture collection	2	1	FDAG69	orf19.3456 p.T148M	
5	SJ01 (Clade III)	Bing et al. 2023[2]	2	2	FDAG41	HYM1 p.E324D	
					FDAG62	NIP100 p.L464*	Figure S1,S8,S10
6	SJ02RE (Clade III)	Bing et al. 2023[2]	2	1	FDAG96	IQG1 p.R569*	
7	RICU4 (Clade III)	Tian et al. 2018[3]	2	1	FDAG63	VRG4 p.48 insert VTNKYVLG	
8	RICU2 (Clade III)	Tian et al. 2018[3]	2	0	NA	NA	NA
9	BJCA002 (Clade III)	Fan et al. 2021[4]	2	0	NA	NA	NA
10	AR0386 (Clade IV)	CDC AR-Bank	4	17	FDAG37	ACE2 p.1-94 loss	Figure S1,S3,S8,S10
					FDAG5	APL2 p.388fs	Figure S1,S8,S10
					FDAG53	APL4 p.436fs	
					FDAG23	APM1 loss	
					FDAG24	APM1 loss	
					FDAG16	ARC19 p.31fs	
					FDAG12	ARV1 p.157fs	
					FDAG39	CAS4 p.L678*	
					FDAG38	CAS4 p.435fs	
					FDAG13	CHS1 p.652fs	Figure S3
					FDAG6	DIG2 p.Y226*	
					FDAG7	DIG2 p.Y226*	
					FDAG99	HOF1 p.Q87*	
					FDAG100	HOF1 p.Q87*	
					FDAG15	KTR2 p.R199T	
					FDAG64	SGS1 p.724fs	
					FDAG28	VAN1 p.410fs	Figure S1,S3,S8,S10
11	XM1805 (Clade III)	Bing et al., 2022[5]	2	0	NA	NA	NA

Notes: Totally, 28 mice were infected with 11 parental strains. Fungal cells were recovered from the brain, liver, spleen, lung, and kidney tissues of these mice after three days of infection. 96 evolved aggregative mutants were identified.

Table S2. Isolated aggregated mutant strains from the mouse infection system (from the brain tissue only).

Parental strain	Source	Number of Infected mice	Number of evolved mutants isolated	Name of isolate	Genotype
BJCA001 (Clade I)	Wang et al. 2018[1]	12	17	FDAG134	ACE2 p.N35fs
				FDAG135	ACE2 p.N35fs
				FDAG136	ACE2 p.N35fs
				FDAG137*	ACE2 p.N35fs
				FDAG138*	ACE2 p.N35fs
				FDAG139	APM1 p.M1K
				FDAG140*	APM1 p.M1K
				FDAG144	CBK1 p.G489R
				FDAG145*	CBK1 p.G489R
				FDAG141	CHS1 p.Y325*
				FDAG142	CHS1 p.Y325*
				FDAG143*	CHS1 p.Y325*
				FDAG150	KRE5 p.K772fs
				FDAG149	LRG1 p.Q93*
				FDAG146	MOB2 p.Q135*
				FDAG147*	MOB2 p.Q135*
				FDAG148	MNN11 p.E116*

Notes: 12 mice were infected with strain BJCA001. Fungal cells were recovered from the brain tissue after three days of infection. 17 evolved aggregative mutant strains were identified. * Identified from the medium without phloxine B. This table is associated with **Dataset S1**.

Table S3. Functional comparison of the mutated genes identified in this study among *C. auris, C. albicans* and *S. cerevisiae.*

Genes	*Phenotype (reference)*		
	C. auris	***C. albicans***	***S. cerevisiae***
ACE2	Aggregates	Filament & Aggregates (Song et al. 2008; Sudbery 2011)[6, 7]	Aggregates (Colman-Lerner et al. 2001)[8]
APL2	Aggregates	Unknown	vacuolar morphology: abnormal (Rad et al. 1995)[9]
APL4	Aggregates	Unknown	vacuolar morphology: abnormal (Kirchhausen 2000)[10]
APM1	Aggregates	Filament increase (Uhl et al. 2003)[11]	vacuolar transport: abnormal (Stepp et al. 1995)[12]
ARC19	Aggregates	Unknown	actin cytoskeleton morphology: abnormal (Winter et al. 1999)[13]
ARC40	Aggregates	Filament increase (Uhl et al. 2003)[11]	actin cytoskeleton morphology: abnormal (Winter et al. 1999)[13]
ARV1	Aggregates	Resistance to drug: decreased (Gallo-Ebert et al. 2012)[14]	GPI proteins transport Defects (Kajiwara et al. 2008)[15]
BNI1	Aggregates	Filamentous growth: decreased (Li et al. 2005)[16]	Polarity defects (Liu et al. 2010)[17]
BSP1	Aggregates	Unknown	Actin dynamics abnormal (Wright et al. 2008)[18]
CAS4	Aggregates	Filament & Aggregates (Song et al. 2008; Sudbery 2011)[6, 7]	Aggregates (Nelson et al. 2003)[19]
CBK1	Aggregates	Filament & Aggregates (Song et al. 2008; Sudbery 2011)[6, 7]	Aggregates (Nelson et al. 2003)[19]
CHS1	Aggregates	Inviable	Aggregates (Oh et al. 2012)[20]
CHS3	Aggregates	Less chitin synthesis in hyphae (Sudoh et al. 1999)[21]	Aggregates (Shaw et al. 1991)[22]
CHS4	Aggregates	Less chitin synthesis in hyphae	Less chitin synthesis in hyphae

		(Sudoh et al. 1999)[21]	(Ono et al. 2000)[23]
CHS6	Aggregates	Unknown	Morphological abnormal (Ziman et al. 1998)[24]
DIG2	Aggregates	Inviable	dig2 dig1 double mutant showed aggregates (Tedford et al. 1997)[25]
ENG1	Aggregates	Cytokinesis: abnormal (Esteban et al. 2005)[26]	Cytokinesis: abnormal (Baladrón et al. 2002)[27]
ENO1	Aggregates	Unknown	Unknown
HOF1	Aggregates	Aggregates (Feng et al. 2020)[28]	Cytokinesis: abnormal (Garabedian et al. 2020)[29]
HSL1	Aggregates	Filament increased (Wightman et al. 2004)[30]	Cell shape: abnormal (Longtine et al. 2000)[31]
HYM1	Aggregates	Filament & Aggregates (Song et al. 2008; Sudbery 2011)[6, 7]	Aggregates (Nelson et al. 2003)[19]
INP52	Aggregates	Unknown	Vacuolar abnormal (Stefan et al. 2005)[32]
IQG1	Aggregates	Cytokinesis: abnormal (Li et al. 2008)[33]	Cytokinesis: abnormal (Osman et al. 2002)[34]
KIC1	Aggregates	Filament & Aggregates (Song et al. 2008; Sudbery 2011)[6, 7]	Aggregates (Nelson et al. 2003)[19]
KIN3	Aggregates	Septin distribution: abnormal (Blankenship et al. 2010)[35]	Unknown
KRE5	Aggregates	Aggregates (Herrero et al. 2004)[36]	Aggregates (Meaden et al. 1990)[37]
KTR2	Aggregates	Unknown	Unknown
LAA1	Aggregates	Unknown	Unknown
LMO1	Aggregates	Filament decreased (Hope et al. 2010)[38]	Resistance to cell wall stress increase (Schmitz et al. 2015)[39]
LRG1	Aggregates	Filament increase (Xie et al. 2016)[40]	Cell fusion: decreased (Fitch et al. 2004)[41]
MNN11	Aggregates	Aggregates (Dean et al. 2022)[42]	Unknown
MOB2	Aggregates	Aggregates (Song et al. 2008)[7]	Aggregates (Nelson et al. 2003)[19]
MYO5	Aggregates	Filament decreased (Oberholzer et al. 2002)[43]	Actin cytoskeleton: abnormal (Moseley et al. 2006)[44]
NIP100	Aggregates	Filament increase (Finley et al. 2008)[45]	Spindle abnormal (Drees et al. 2001)[46]

OCH1	Aggregates	Aggregates (Bates et al. 2006)[47]	Aggregates (Mondesert et al. 1997)[48]
orf19.3456	Aggregates	Unknown	Unknown
PAN1	Aggregates	Unknown	Actin cytoskeleton: abnormal (Moseley et al. 2006)[44]
PEX5	Aggregates	Unknown	Unknown
SAC6	Aggregates	Filament increase (Zhang et al. 2016)[49]	Actin cytoskeleton: abnormal (Moseley et al. 2006)[44]
SGS1	Aggregates	Unknown	Unknown
SLA2	Aggregates	Filament absent (Gale et al. 2009)[50]	Actin cytoskeleton: abnormal (Moseley et al. 2006)[44]
SOG2	Aggregates	Filament & Aggregates (Song et al. 2008；Sudbery 2011)[6, 7]	Aggregates (Nelson et al. 2003)[19]
SPA2	Aggregates	Filamentous growth: decreased (Zheng et al. 2003)[51]	Polarity defects (Liu et al. 2010)[17]
VAN1	Aggregates	Aggregates (Dean et al. 2022)[42]	Unknown
VRG4	Aggregates	inviable	inviable

Table S4. Isolated aggregated mutant strains from the antimicrobial peptide assay.

No.	Parental strain	Reference or source	Number of evolved mutants	Name of isolate	Genotype	Used in this article
1	BJCA001 (Clade I)	Wang et al. 2018[1]	18	FDAG104	ACE2 p.D521delete	Figure S12
				FDAG117	CAS4 p.E114*	
				FDAG121	CAS4 p.R1263K	
				FDAG124	CAS4 p.Y1113*	
				FDAG125	CAS4 p.2132fs	
				FDAG126	CAS4 p.1301fs	
				FDAG101	CBK1 p.375fs	Figure S12
				FDAG130	CHS1 p.P538L	
				FDAG119	CHS4 p.S145*	
				FDAG113	CHS6 p.Y85*	
				FDAG105	ENG1 p.Y598*	
				FDAG120	ENO1 p.A110V	
				FDAG133	INP52 p.Q547*	
				FDAG114	LAA1 p.LF1021QI	Figure S12
				FDAG122	LAA1 p.837fs	
				FDAG127	LAA1 p.490fs	
				FDAG118	SLA2 p.865fs	
				FDAG123	VRG4 p.M252R	
2	CBS12766 (Clade I)	CBS-KNAW culture collection	1	FDAG106	KIC1 p.E452*	
3	CBS10913 (Clade II)		3	FDAG131	CBK1 p.E601V	
				FDAG132	INP52 p.Q426*	
				FDAG112	PEX5 p.E209*	
4	SJ01 (Clade III)	Bing et al. 2023[2]	2	FDAG116	SOG2 p.444fs	
				FDAG103	SPA2 p.Q403*	Figure S12
5	RICU2 (Clade III)	Tian et al. 2018[3]	1	FDAG115	LMO1 p.S54V	
6	BJCA002 (Clade III)	Fan et al. 2021[4]	3	FDAG107	LRG1 p.544fs	
				FDAG110	MYO5 p.1118fs	
				FDAG111	SLA2 p.498fs	
7	AR0386 (Clade IV)	CDC AR-Bank	5	FDAG102	ARC40 p.S263*	
				FDAG109	CHS3 p.373fs	Figure S12
				FDAG128	HYM1 p.33fs	
				FDAG129	SAC6 p.472fs	
				FDAG108	VAN1 p.R228*	

Notes: Seven clinical strains of different genetic clades were treated with the human antimicrobial peptides LL-37 and PACAP.

References:

1. Wang XJ*, et al.* The first isolate of Candida auris in China: clinical and biological aspects. *Emerg Microbes Infec* **7**, 93 (2018).
2. Bing J*, et al.* Clinical isolates of Candida auris with enhanced adherence and biofilm formation due to genomic amplification of ALS4. *PLoS pathogens* **19**, e1011239 (2023).
3. Tian S*, et al.* First cases and risk factors of super yeast Candida auris infection or colonization from Shenyang, China. *Emerg Microbes Infec* **7**, 128 (2018).
4. Fan SR*, et al.* A biological and genomic comparison of a drug-resistant and a drug-susceptible strain of Candida auris isolated from Beijing, China. *Virulence* **12**, 1388-1399 (2021).
5. Bing J*, et al.* A case of Candida auris candidemia in Xiamen, China, and a comparative analysis of clinical isolates in China. *Mycology* **13**, 68-75 (2022).
6. Sudbery PE. Growth of Candida albicans hyphae. *Nature reviews Microbiology* **9**, 737-748 (2011).
7. Song Y*, et al.* Role of the RAM Network in Cell Polarity and Hyphal Morphogenesis in Candida albicans. *Molecular Biology of the Cell* **19**, 5456-5477 (2008).
8. Colman-Lerner A, Chin TE, Brent R. Yeast Cbk1 and Mob2 activate daughter-specific genetic programs to induce asymmetric cell fates. *Cell* **107**, 739-750 (2001).
9. Rad MR*, et al.* Saccharomyces-Cerevisiae Apl2p, a Homolog of the Mammalian Clathrin Ap Beta-Subunit, Plays a Role in Clathrin-Dependent Golgi Functions. *Journal of Cell Science* **108**, 1605-1615 (1995).
10. Kirchhausen T. Three ways to make a vesicle. *Nat Rev Mol Cell Bio* **1**, 187-198 (2000).
11. Uhl MA, Biery M, Craig N, Johnson AD. Haploinsufficiency-based large-scale forward genetic analysis of filamentous growth in the diploid human fungal pathogen C.albicans. *Embo Journal* **22**, 2668-2678 (2003).
12. Stepp JD, Pellicenapalle A, Hamilton S, Kirchhausen T, Lemmon SK. A Late Golgi Sorting Function for Saccharomyces-Cerevisiae Apm1p, but Not for Apm2p, a 2nd Yeast Clathrin Ap Medium Chain-Related Protein. *Molecular Biology of the Cell* **6**, 41-58 (1995).
13. Winter DC, Choe EY, Li R. Genetic dissection of the budding yeast Arp2/3 complex:: A comparison of the and structural roles of individual subunits. *P Natl Acad Sci USA* **96**, 7288-7293 (1999).
14. Gallo-Ebert C*, et al.* Arv1 lipid transporter function is conserved between pathogenic and nonpathogenic fungi. *Fungal genetics and biology* **49**, 101-113 (2012).
15. Kajiwara K*, et al.* Yeast ARV1 is required for efficient delivery of an early GPI intermediate to the first mannosyltransferase during GPI assembly and controls lipid flow from the endoplasmic reticulum. *Molecular Biology of the Cell* **19**, 2069-2082 (2008).
16. Li CR, Wang YM, Zheng XD, Liang HY, Tang JCW, Wang Y. The formin family protein CaBni1p has a role in cell polarity control during both yeast and hyphal growth in Candida albicans. *Journal of Cell Science* **118**, 2637-2648 (2005).
17. Liu BD*, et al.* The Polarisome Is Required for Segregation and Retrograde Transport of Protein Aggregates. *Cell* **140**, 257-267 (2010).
18. Wright DJ*, et al.* The actin cytoskeletal component Bsp1p has an auxiliary role in

actomyosin ring function and in the maintenance of bud-neck structure. *Genetics* **178**, 1903-1914 (2008).
19. Nelson B*, et al.* RAM: A conserved signaling network that regulates Ace2p transcriptional activity and polarized morphogenesis. *Molecular Biology of the Cell* **14**, 3782-3803 (2003).
20. Oh Y, Chang KJ, Orlean P, Wloka C, Deshaies R, Bi EF. Mitotic exit kinase Dbf2 directly phosphorylates chitin synthase Chs2 to regulate cytokinesis in budding yeast. *Molecular Biology of the Cell* **23**, 2445-2456 (2012).
21. Sudoh M*, et al.* The Candida albicans CHS4 gene complements a mutation and is involved in chitin biosynthesis. *Microbiol-Uk* **145**, 1613-1622 (1999).
22. Shaw JA*, et al.* The function of chitin synthases 2 and 3 in the Saccharomyces cerevisiae cell cycle. *J Cell Biol* **114**, 111-123 (1991).
23. Ono N*, et al.* The yeast Chs4 protein stimulates the trypsin-sensitive activity of chitin synthase 3 through an apparent protein-protein interaction. *Microbiol-Uk* **146**, 385-391 (2000).
24. Ziman M, Chuang JS, Tsung M, Hamamoto S, Schekman R. Chs6p-dependent anterograde transport of Chs3p from the chitosome to the plasma membrane in. *Molecular Biology of the Cell* **9**, 1565-1576 (1998).
25. Tedford K, Kim S, Sa D, Stevens K, Tyers M. Regulation of the mating pheromone and invasive growth responses in yeast by two MAP kinase substrates. *Current Biology* **7**, 228-238 (1997).
26. Esteban PF*, et al.* Characterization of the gene encoding an endo-1,3-β-glucanase involved in cell separation in. *Current Microbiology* **51**, 385-392 (2005).
27. Baladrón V, Ufano S, Dueñas E, Martín-Cuadrado AB, del Rey F, de Aldana CRV. Eng1p, an endo-1,3-β-glucanase localized at the daughter side of the septum, is involved in cell separation in. *Eukaryotic Cell* **1**, 774-786 (2002).
28. Feng JR*, et al.* Hof1 plays a checkpoint-related role in MMS-induced DNA damage response in. *Molecular Biology of the Cell* **31**, 348-359 (2020).
29. Garabedian MV, Wirshing A, Vakhrusheva A, Turegun B, Sokolova OS, Goode BL. A septin-Hof1 scaffold at the yeast bud neck binds and organizes actin cables. *Molecular Biology of the Cell* **31**, 1988-2001 (2020).
30. Wightman R, Bates S, Amornrrattanapan P, Sudbery P. In Candida albicans, the Nim1 kinases Gin4 and Hsl1 negatively regulate pseudohypha formation and Gin4 also controls septin organization. *Journal of Cell Biology* **164**, 581-591 (2004).
31. Longtine MS, Theesfeld CL, McMillan JN, Weaver E, Pringle JR, Lew DJ. Septin-dependent assembly of a cell cycle-regulatory module in. *Molecular and Cellular Biology* **20**, 4049-4061 (2000).
32. Stefan CJ, Padilla SM, Audhya A, Emr SD. The phosphoinositide phosphatase Sjl2 is recruited to cortical actin patches in the control of vesicle formation and fission during endocytosis. *Molecular and Cellular Biology* **25**, 2910-2923 (2005).
33. Li CR, Wang YM, Wang Y. The IQGAP Iqg1 is a regulatory target of CDK for cytokinesis in. *Embo Journal* **27**, 2998-3010 (2008).
34. Osman MA, Konopka JB, Cerione RA. Iqg1p links spatial and secretion landmarks to polarity and cytokinesis. *Journal of Cell Biology* **159**, 601-611 (2002).

35. Blankenship JR, Fanning S, Hamaker JJ, Mitchell AP. An Extensive Circuitry for Cell Wall Regulation in. *PLoS pathogens* **6**, e1000752 (2010).
36. Herrero AB, Magnelli P, Mansour MK, Levitz SM, Bussey H, Abeijon C. KRE5 gene null mutant strains of Candida albicans are avirulent and have altered cell wall composition and hypha formation properties. *Eukaryot Cell* **3**, 1423-1432 (2004).
37. Meaden P, Hill K, Wagner J, Slipetz D, Sommer SS, Bussey H. The yeast KRE5 gene encodes a probable endoplasmic reticulum protein required for (1----6)-beta-D-glucan synthesis and normal cell growth. *Mol Cell Biol* **10**, 3013-3019 (1990).
38. Hope H, Schmauch C, Arkowitz RA, Bassilana M. The Candida albicans ELMO homologue functions together. *Molecular Microbiology* **76**, 1572-1590 (2010).
39. Schmitz HP, Jendretzki A, Wittland J, Wiechert J, Heinisch JJ. Identification of Dck1 and Lmo1 as upstream regulators of the small GTPase Rho5 in. *Molecular Microbiology* **96**, 306-324 (2015).
40. Xie JL, Grahl N, Sless T, Leach MD, Kim SH, Hogan DA, Robbins N, Cowen LE. Signaling through Lrg1, Rho1 and Pkc1 Governs Morphogenesis in Response to Diverse Cues. *Plos Genetics* **12**, e1006405 (2016).
41. Fitch PG, Gammie AE, Lee DJ, de Candal VB, Rose MD. Lrg1p is a Rho1 GTPase-activating protein required for efficient cell fusion in yeast. *Genetics* **168**, 733-746 (2004).
42. Dean N, Jones R, DaSilva J, Chionchio G, Ng H. The Mnn10/Anp1-dependent N-linked outer chain glycan is dispensable for Candida albicans cell wall integrity. *Genetics* **221**, iyac048 (2022).
43. Oberholzer U, Marcil A, Leberer E, Thomas DY, Whiteway M. Myosin I is required for hypha formation in. *Eukaryotic Cell* **1**, 213-228 (2002).
44. Moseley JB, Goode BL. The yeast actin cytoskeleton: From cellular function to biochemical mechanism. *Microbiol Mol Biol R* **70**, 605-45 (2006).
45. Finley KR, Bouchonville KJ, Quick A, Berman J. Dynein-dependent nuclear dynamics affect morphogenesis in Candida albicans by means of the Bub2p spindle checkpoint. *J Cell Sci* **121**, 466-476 (2008).
46. Drees BL*, et al.* A protein interaction map for cell polarity development. *Journal of Cell Biology* **154**, 549-571 (2001).
47. Bates S*, et al.* Outer chain N-glycans are required for cell wall integrity and virulence of. *Journal of Biological Chemistry* **281**, 90-98 (2006).
48. Mondesert G, Clarke DJ, Reed SI. Identification of genes controlling growth polarity in the budding yeast Saccharomyces cerevisiae: A possible role of N-glycosylation and involvement of the exocyst complex. *Genetics* **147**, 421-434 (1997).
49. Zhang B*, et al.* The Candida albicans fimbrin Sac6 regulates oxidative stress response (OSR) and morphogenesis at the transcriptional level. *Biochim Biophys Acta* **1863**, 2255-2266 (2016).
50. Gale CA*, et al.* SLA2 mutations cause SWE1-mediated cell cycle phenotypes in Candida albicans and Saccharomyces cerevisiae. *Microbiology (Reading)* **155**, 3847-3859 (2009).
51. Zheng XD, Wang YM, Wang Y. CaSPA2 is important for polarity establishment and maintenance in Candida albicans. *Molecular Microbiology* **49**, 1391-1405 (2003).

Dataset S1. Detailed information for the evolved aggregative isolates and deletion mutant strains used in this study.

Dataset S2. SRA accession numbers for the *C. auris* genomic sequences and RNA-Seq data from this study, publicly available genomic sequences, and mutational analyses.

Dataset S3. RNA-seq data of the yeast-form and aggregative isolates.

Dataset S4. Primers used in this study.
