# Supplementary material for: Rapid evolution of an adaptive multicellular morphology of Candida auris during systemic infection
Source: Nat Commun. 2024 Mar 16;15:2381. doi: 10.1038/s41467-024-46786-8 (PMC10944540; doi:10.1038/s41467-024-46786-8)
Supplement: Supplementary file 3 — Description of Additional Supplementary Files [file 41467_2024_46786_MOESM3_ESM.pdf]

Description of Additional Supplementary Files

File Name: Dataset S1
Description: Detailed information for the evolved aggregative isolates and deletion mutant strains used in this study

File Name: Dataset S2
Description: SRA accession numbers for the *C. auris* genomic sequences and RNA-Seq data from this study, publicly available genomic sequences, and mutational analyses.

File Name: Dataset S3
Description: RNA-seq data of the yeast-form and aggregative isolates.

File Name: Dataset S4
Description: Primers used in this study.
